# Effects of Electromagnetically Treated Water (EMTW) on the Properties of Water and Photosynthetic Performance of *Spinacia oleracea* L.

**DOI:** 10.3390/plants14192972

**Published:** 2025-09-25

**Authors:** Lyubka Koleva-Valkova, Ignat Ignatov, Fabio Huether, Bojin Bojinov, Kiril Marinkov, Teodora P. Popova, Alexander I. Ignatov, Yordan G. Marinov, Mario T. Iliev

**Affiliations:** 1 Department of Plant Physiology, Biochemistry and Genetics, Agricultural University, 4000 Plovdiv, Bulgaria; bojinov@au-plovdiv.bg (B.B.); kmarinkov@au-plovdiv.bg (K.M.); 2 Scientific Research Center of Medical Biophysics (SRCMB), 1111 Sofia, Bulgaria; alexander.ignatov06@gmail.com; 3 EVODROP AG, 8306 Brüttisellen, Switzerland; fabio.huether@evodrop.com; 4 Faculty of Veterinary Medicine, University of Forestry, 10 Kl. Ohridski Blvd., 1756 Sofia, Bulgaria; dr_tpopova@abv.bg; 5 Georgi Nadjakov Institute of Solid State Physics, Bulgarian Academy of Sciences, 72 Tzarigradsko Chaussee Blvd., 1784 Sofia, Bulgaria; ymarinov@issp.bas.bg; 6 Faculty of Physics, Sofia University, St. Kliment Ohridski, 1000 Sofia, Bulgaria; ozo@phys.uni-sofia.bg

**Keywords:** EMTW, irrigation, photosynthesis, phenols, flavonoids, spinach, ^1^H-NMR, DFT

## Abstract

The applications of electromagnetic (EM) field treatment on water in agriculture have garnered increasing attention as a sustainable method to enhance plant growth, water-use efficiency, and metabolic performance. A growing body of evidence suggests that exposure to EM fields can affect water molecules, possibly by influencing hydrogen bonding dynamics, the structuring of water clusters, and electrokinetic properties of the water molecules. These alterations are thought to correlate with plant physiological performance. The methodology of the study was divided into two parts. The first part focused on the preparation of electromagnetically treated water. The second part involved applying this treated water to spinach plants. The present study investigates the physiological responses of *Spinacia oleracea* L. to irrigation with electromagnetically treated water (EMTW), focusing on elucidating the potential mechanisms that may underlie the observed effects. EMTW was generated using a solenoid-based system operating in dual-frequency ranges (100–1000 Hz and 10–100 kHz), which has been previously shown to influence both the microbiological and electrokinetic properties of aqueous systems. To evaluate the structural and functional implications of EMTW, a combined methodological approach was employed, integrating proton nuclear magnetic resonance (^1^H-NMR) spectroscopy, density functional theory (DFT) modeling of water hydrogen bonds and clusters, and comprehensive plant physiological assessments. Plants were cultivated under both controlled and field conditions to assess consistency across environmental settings. Physiological measurements demonstrated that EMTW irrigation increased photosynthetic rate by ~80%, transpiration by 49–67%, stomatal conductance by 78–129%, intercellular CO_2_ concentration by 42–80%, and chlorophyll content by 9.3–9.5% compared to control samples. Additionally, phenoloc and flavonoid contents were elevated by 7.4% and 7.6%, respectively, in field-grown plants. These enhancements were statistically significant (*p* < 0.001 or *p* < 0.01) under both laboratory and field conditions, confirming the robustness of the observed effects.

## 1. Introduction

In recent years, increasing attention has been given to the use of electromagnetic treatments of water, in which the water is simultaneously exposed to both electric and magnetic field components. According to Maxwell’s equations, electric and magnetic fields are oriented in mutually perpendicular planes and are both perpendicular to the direction of wave propagation. While permanent magnetic fields have been shown to alter the surface tension and influence the hydrogen network and the clustering structuring of water molecules, the addition of an electric field enhances these effects by altering electrokinetic properties and ion mobility. As a result, more stable changes in the physicochemical properties of water are achieved, which positively affect nutrient uptake, photosynthetic activity, and plant growth.

Magnetic and electromagnetic fields have been increasingly explored for their potential to alter the physicochemical properties of water, with an impact on agricultural applications. The influence of water extends to molecular restructuring, hydrogen bonding, and changes in physicochemical parameters, such as surface tension and electrical conductivity. By modifying water’s biological activity, these fields can potentially enhance plant systems, improving crop production.

Magnetic and electromagnetic fields exert notable effects on the structure of water, primarily by influencing hydrogen bonding and molecular restructuring. Research on the effects of a magnetic field was achieved with infrared (IR), UV–visible spectroscopy, Raman, and XRD [1]. In this research, it was proven that alteration in spectral characteristics (UV-Vis, IR, Raman, XRD) reduced surface tension, increased molecular polarization, and induced structural changes in water clusters with more stable hydrogen bond configurations with saturation of memory effects. The authors investigate the effects of a magnetic field on water, including its impact on surface tension, viscosity, evaporation rate, and adhesion. They emphasize that the effects depend on parameters such as field intensity and gradient, and that some changes persist even after the magnetic field is removed [2]. Studies have shown that exposure to a permanent magnetic field with magnetic induction 0.3 T or 3000 G results in changes in the properties of water [3]. Exposure to a magnetic field with an induction of 3000 G alters the molecular properties of hydrogen bonding, potentially affecting pH and the electrical conductivity of solution of potassium carbonate [4,5].

The following results were obtained using magnetically treated water (MTW) exposed to a permanent magnetic field. The results of treatment with MTW were observed in terms of seed germination [6]. Exposure of maize (*Zea mays*) seeds to a permanent magnetic field of 0.15 T for 10 min resulted in increased germination rate, shoot length, fresh biomass, as well as a ~25% rise in the specific electrical conductivity of the seed water extract [7]. Studies have shown that MTW can increase the rates of photosynthesis, transpiration, and stomatal conductance in various plant species, including pepper [8], *Glycine max*, *Triticum aestivum*, *Nicotiana tabacum*, and *Lactuca sativa* [9]. An increase in photosynthetic pigments, such as chlorophyll a, chlorophyll b, and carotenoids, has also been observed, which is a prerequisite for enhanced light energy absorption and increased photosynthesis [10]. The application of magnetically treated water affects the chlorophyll a/b ratio, which affects the activity of light-harvesting complex II (LHC II) [11].

The application of MTW is associated with changes in the physicochemical properties of water that contribute to its impact on processes within the plant organism [12,13].

Electric and magnetic fields influence the structure of water. Electric fields influence the orientation of water dipoles, the reorganization of hydrogen bonds, and alterations in dielectric properties. Magnetic fields affect the hydrogen-bonding network, forming water clusters [14].

The results are presented for the electromagnetic (EM) treatment of saline water, which impacts crop performance and soil properties. Researchers have tested this on crops such as maize and potatoes. EM-treated water improved seed germination and enhanced early plant growth in maize. Potato plants irrigated with treated water showed a yield increase of about 10%. The treatment helped leach salts deeper into the soil, reducing salinity around the root zone. This approach provides a sustainable solution for utilizing saline water in agriculture, especially in areas with freshwater scarcity [15].

Researchers investigated the effects of electromagnetically treated irrigation water on potato growth under saline conditions. Salinity stress is known to impair plant metabolism and significantly reduce crop yields. The study demonstrated that electromagnetic treatment of water enhanced photosynthetic pigment concentration, enzymatic activity, and water-use efficiency in potato plants. These improvements influence and mitigate the negative effects of salt stress. As a result, treated plants showed improved physiological performance and significantly higher tuber yields compared to untreated controls. The findings suggest that electromagnetic water treatment is a promising and sustainable agronomic approach for enhancing crop productivity in saline environments [16].

Alternating electric fields accelerate the molecular dynamics of water molecules. Notably, the described effects pertain to rewiring and diffusion [17]. Research provides experimental evidence that external electric fields cause macroscopic alignment of water molecules’ dipoles, forming columns around 60 µm long. The measurements conform to a state of highly aligned dipoles within each column [18].

Several studies have established positive effects of electromagnetically treated water (EMTW) on various physicochemical and biochemical processes in plants. The results show that the treatment on cotton increases dry matter accumulation by 12.7–52.2%, cotton seed yield by 30.8–38.7%, and harvest index to 0.33 [19]. The effect of EMAW on plant cells is associated with changes in the activity of some antioxidant enzymes and the maintenance of photosynthetic membranes, indicating a multifaceted mechanism of action [20]. Interesting results were achieved using water treated with magneto-hydrodynamic forces on *Haberlea rhodopensis*. They were studied in terms of the restructuring of the hydrogen network of water molecules and the formation of clusters [21]. A study revealed that during the drying process in *H. rhodopensis*, the number of free water molecules decreases, and water dimers are formed [22].

EMTW within the applied frequency ranges of 100–1000 Hz and 10–100 kHz has been shown to modify hydration parameters [23]. In vitro assays using *Brassica oleracea* L. var. italica demonstrated that EMTW exhibited enhanced antimicrobial activity, suggesting potential bioprotective applications. These findings support a possible influence on water influx and plant hydration dynamics.

Despite these promising reports, the mechanisms through which electromagnetic treatment of water influences plant physiology require further investigation. This study aims to determine whether electromagnetic water treatment can enhance plant physiological performance via alterations in water parameters.

## 2. Results

The control water and EMTW for irrigation correspond to the Bulgarian Ordinance No. 18 on the quality of water for irrigation of agricultural crops [24,25].

### 2.1. Nuclear Magnetic Resonance (NMR)

**Figure 1:**
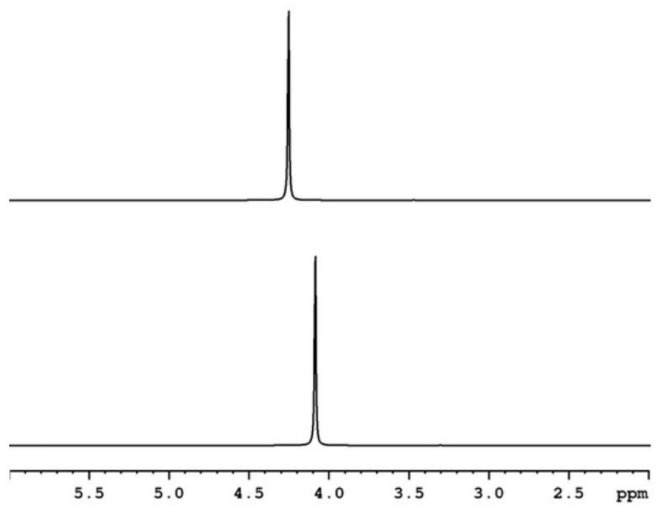
^1^H-NMR of the sample of EMT irrigation water (**top**) and the control sample (**bottom**).

Figure 1 shows ^1^H-NMR [26] of the control sample and the sample of EMTW for irrigation.

Table 1 presents the δ values, in parts per million (ppm), indicating changes in the chemical environment of nuclei. The Δν_1/2_, Hz values, reflecting the width of the resonance peak at half-height, are associated with the molecular motion and restructuring of water molecules.

The 1H-NMR analysis revealed measurable changes in water after electromagnetic treatment. Specifically, the proton signal of treated water shifted downfield by Δδ = 0.061 ppm (sample relative to control), and the peak half-height width (Δν_1/2_) broadened 0.59 Hz (Table 1). The biological implications of these spectral changes require cautious interpretation and further replication.

### 2.2. DFT-Computed Averaged NMR Chemical Shifts on the Size of the Modeled Clusters and Hydrogen Bonds

**Figure 2:**
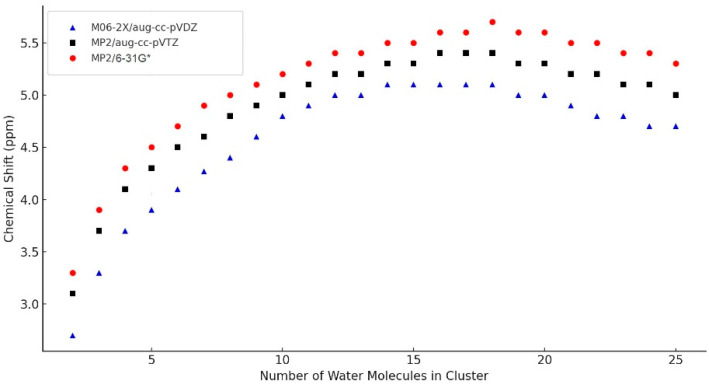
Correlation between the number of water molecules in (H_2_O)_n_ clusters and their NMR chemical shifts (δ, ppm).

DFT modeling was used to explore the relationship between water cluster size and NMR chemical shift [26,27,28]. Figure 2 demonstrates that as the water cluster size increases from *n* = 2 to *n* = 16 molecules, the calculated chemical shift (δ) follows a logarithmic growth trend, which reaches a plateau at a larger cluster size for *n* > 16. For the values from *n* = 2 to *n* = 4 the trend is exponential.

Figure 2 compares the correlation between the number of water molecules in (H_2_O)_n_ clusters and their NMR chemical shifts (δ, ppm).

**Figure 3:**
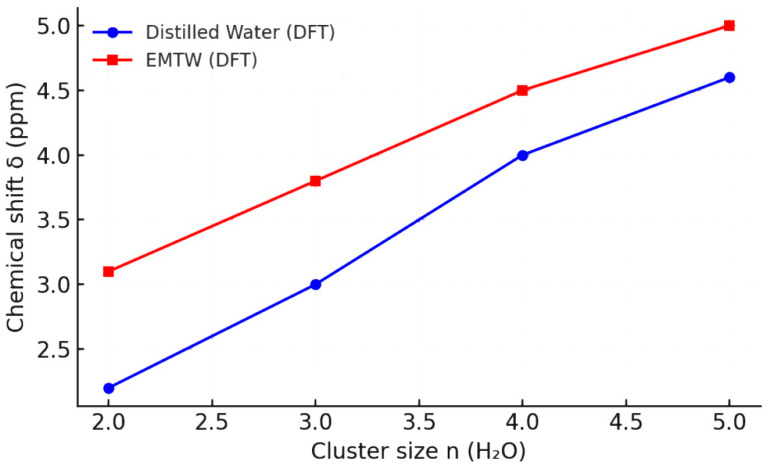
Dependence of the DFT-calculated chemical shifts (δ, ppm) on the size of water clusters for distilled water and EMTW.

Figure 3 demonstrates the dependence of the DFT-calculated chemical shifts (δ, ppm) on the size of water clusters for distilled water and EMTW.

The data show that as the number of molecules in a water cluster increases, the chemical shift rises for both types of samples. EMTW consistently exhibits higher values. For the range *n* = 2–4, an exponential growth trend is observed, whereas for *n* = 2–5, the relationship follows a logarithmic pattern, and these calculations were performed with Residual Sum Squares (RSS) for MP2/aug-cc-pVTZ. This indicates greater polarization and ordering of the hydrogen-bond network in EMTW. This type of modeling, which incorporates linear, logarithmic, and exponential trends in chemical shifts as a function of cluster sizes, was demonstrated by Ignatov et al. in 2025 [27].

Water clusters with chemical formulas (H_2_O)_n=2,4,6,8p10_ are visually presented in [28]. In EMTW, a pronounced hydrogen-bond dynamics is observed in the structuring of clusters up to pentamers, which is typical for the liquid phase of water and its solvent-like behavior [27,29,30].

There is a proportional relationship between the number of molecules in water clusters and the number of hydrogen bonds. This analysis indicates that in the biologically relevant aqueous system, such as EMTW, there is structuring of hydrogen bonds and water clusters. This structural network may contribute to observed physiological responses.

The EMTW is associated with model-based predictions of enhanced hydrogen-bond ordering in small-scale water clusters with higher dipole moments and elevated chemical shifts (δ) compared to distilled water [27] or a control sample with irrigation water. It reaches a logarithmic stabilization δ at a lower number of hydrogen bonds. This indicates earlier structural saturation.

The following formulas are valid for the logarithmic trends for *n* = 2–5 [27]:

Distilled water:

δ = 2.668ln(n) + 0.257(1)

Electromagnetically treated water (EMTW):

δ = 2.088ln(n) + 1.601(2)

These structural properties may contribute to the observed physiological responses, though further investigation is required to confirm the causal relationship.

It should be noted that the DFT-calculated water clusters represent idealized laboratory conditions based on samples of the EMTW and control water used for irrigation.

Exploring the properties of these water clusters concerning bulk water behavior in soil–plant systems requires additional experimental validation.

It is well established that divalent ions such as calcium (Ca^2+^) and magnesium (Mg^2+^) contain a first shell with 6–8 water molecules for Ca^2+^, and 6 for Mg^2+^, and up to 24 water molecules in the second hydration shell [29].

EMTW-treated water may exhibit an increased number of hydrogen bonds and more stable water clusters, potentially supporting the stabilization of the hydration shells around calcium and magnesium ions.

Calcium and magnesium ions play a key role in plant metabolism. Calcium is involved in cellular signaling and structural stability. Magnesium is essential for photosynthesis and energy transfer as the central ion in chlorophyll and a cofactor for numerous enzymes.

### 2.3. Physiological and Biochemical Indicators of Spinacia oleracea L.

Table 2 presents the results of the photosynthetic performance and chlorophyll content. Electromagnetically treated water significantly impacts the leaf gas exchange of plants in both experimental settings (laboratory-grown (LG) and field-grown (FG) spinach plants). Plants from group B show almost twice as high photosynthetic activity (an increase of 81.7% (LG) and 80.1% (FG) compared to the control group A). The results show an increase in the transpiration rate by 66.7% (LG) and 48.9% (FG). The data illustrate an increase in stomatal conductance of 129% (LG) and 78% (FG). The experiments show an increase in intercellular CO_2_ concentration of 79.8% (LG) and 42.1% (FG). Higher chlorophyll values are also recorded, and the increases are 9.5% (LG) and 9.3% (FG) compared with the control plants.

Differences between the EMTW and control water were assessed based on physiological measurements of *Spinacia oleracea* L. plants. Key physiological parameters, including photosynthesis, stomatal conductance, transpiration, and chlorophyll content, exhibited strong positive correlations, as indicated by Pearson’s test, with r values ranging from 0.61 to 0.99. Several moderate correlations (e.g., r = 0.48 to 0.74) also suggested a possible association.

The changes are statistically significant (*p* < 0.001 or *p* < 0.01) under both laboratory and field growing conditions. Table 3 demonstrates that the correlation coefficient (r) indicates a strong relationship between the EMTW treatment and physiological response (e.g., r = 0.744) for photosynthesis in laboratory conditions.

The strong positive correlation between photosynthesis and stomatal conductance indicates that EMTW stimulates stomatal opening, allowing for improved gas exchange and increased water flow. This leads to the efficiency of the photosynthetic apparatus, supported by improved CO_2_ uptake and leaf cooling through transpiration.

**Figure 4:**
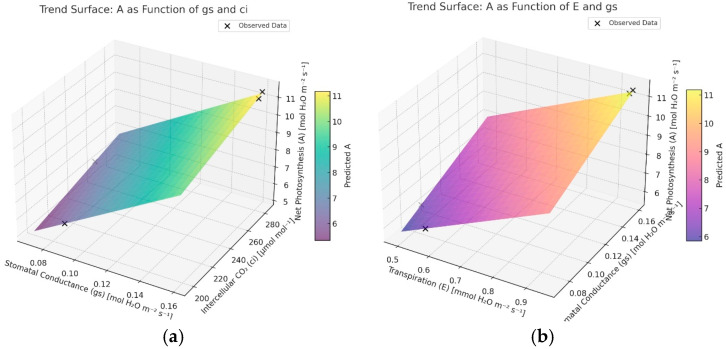
Predicted 3D trend surfaces on A_net_: (**a**) as a function of g_s_ and C_i_; (**b**) as a function of E and g_s_, under EMTW treatment.

Figure 4a shows the predicted 3D surface of net photosynthesis *A_net_* as a function of stomatal conduction *g_s_* and intercellular CO_2_ concentration *g_s_*. Figure 4b illustrates *A_net_* as a function of transpiration E and g_s_ under EMTW irrigation. Warmer colors indicate higher A_net_; black crosses denote observed data points. The surfaces confirm that increases in g_s_ together with E or c_i_ are associated with higher photosynthetic rates, consistent with the strong Pearson correlations in Table 3. The fitted models are as follows:

A_net_ = 0.36 + 7.25E + 25.61g_s_ (3)

A_net_ = −1.89 + 51.04s + 0.02C_i_ (4)

These relationships highlight the central role of stomatal opening and CO_2_ availability in determining photosynthetic efficiency and support the hypothesis that EMTW promotes effective water vapor exchange (transpiration), possibly via increased guard-cell turgor driven by H^+^-ATPase activity, leading to wider stomatal opening.

Figure 4 visualizes predicted 3D trend surfaces on A_net_: (a) as a function of g_s_ and C_i_; (b) as a function of E and g_s_, under the EMTW treatment. Warmer colors indicate higher A_net_; crosses mark observed data.

Table 4 presents the results of the total phenolic and flavonoid content. A modest increase in the content of phenolic compounds (+7.4%) and, specifically, flavonoids (+7.6%) was observed in the field-grown (FG) plant group irrigated with electromagnetically treated water compared to the control plants [31,32].

**Figure 5:**
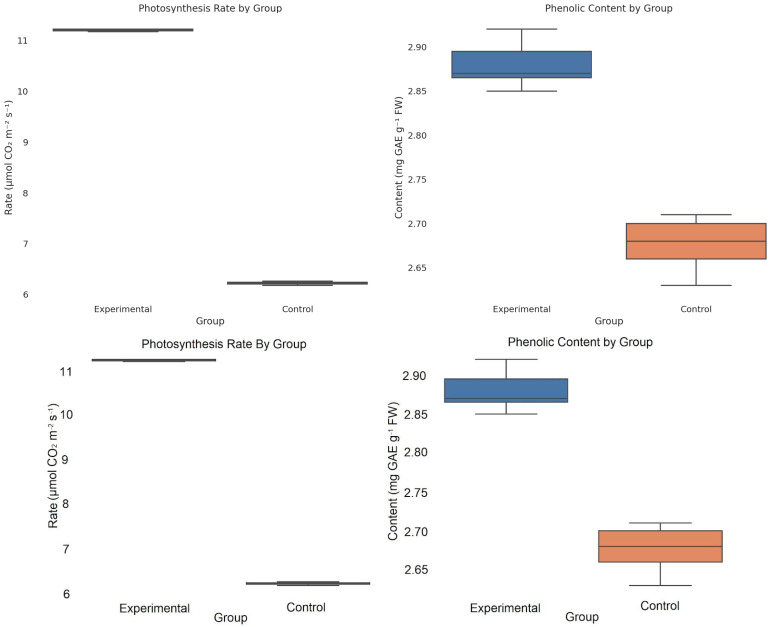
Comparison of mean net photosynthetic rate (A_net_) and total phenolic content in field-grown spinach (FG) under control vs. EMTW irrigation.

Welch’s *t*-test was used to compare the means of two independent groups without assuming equal variances. The test adjusts the degrees of freedom accordingly, making it suitable for biological and agronomic studies. Figure 5 demonstrates the Welch’s *t*-test comparison of means for the net photosynthetic rate (A_net_) and total phenolic content in the FG group.

Figure 5 demonstrates that EMTW significantly increased photosynthetic activity in *Spinacia oleracea* L. compared with the control group (*p* < 0.001). The phenolic content was also higher under the EMTW treatment, suggesting simulation of secondary metabolism or defense-related biochemical pathways. These results indicate a positive effect of EMTW on both the physiological and biochemical parameters of *Spinacia oleracea* L.

## 3. Discussion

The quantum chemical analyses indicate that compared to untreated water, EMTW exhibits a more stable and more polarized hydrogen-bonding network and an altered water cluster structure. The ^1^H NMR results support this, showing a downfield shift in the water proton signal from δ = 4.192 ppm (control sample) to δ = 4.253 ppm (EMTW). In general, a downfield shift is consistent with stronger hydrogen bonding and a larger effective cluster size. Our DFT models predict enhanced hydrogen bonding in EMTW, while cluster growth plateaus at relatively small *n* = 2–6, implying numerous stable small clusters rather than unlimited growth. These molecular features may have physiological implications in plants.

EMTW is associated with changes in several physicochemical parameters beyond hydrogen bonding and water clusters. These include increased electrical conductivity, elevated dipole moment (as predicted by DFT), and a slight shift in pH.

Theoretical models suggest that enhanced dipole polarization and altered electrokinetic properties may facilitate different interactions with ions and biomolecules, possibly influencing ion transport and stomatal regulation.

Beyond hydrogen bonding and cluster structuring, EMTW showed changes in several physicochemical parameters: higher electrical conductivity, elevated dipole moments (DFT predictions), and a slight pH shift.

In our spinach experiments, it was associated with strong positive physiological effects, including higher net photosynthesis (A_net_), greater stomatal conductance (g_s_), and increased intercellular CO_2_(C_i_) (Table 2). The high correlations between the parameters indicate coordinated improvements, suggesting a systemic enhancement of water and carbon metabolism. As noted in [33], stomatal opening depends on osmotic gradients generated by H^+^-ATPase activation and subsequent K^+^ accumulation in guard cells. Consistent with this framework, EMTW may facilitate gas exchange mechanisms and help improve water uptake and stomatal function, leading to the observed increases in photosynthetic parameters.

Electromagnetically treated water exhibited increased electrical conductivity [23], a parameter relevant to osmotic behavior and ion transport in the plant-water system.

Our integrated study, combining NMR, DFT, and plant physiological assessments, provides a framework for understanding EMTW effects in *Spinacia oleracea* L. Modeling approaches such as those in [34] support the use of DFT/continuum solvation methods to probe dipolar polarization and stability in aqueous systems.

Theoretical modeling of hydrogen-bonded water clusters using DFT provides insights into energetics and dipole properties relevant to EMTW.

Under both the field and laboratory conditions, the agro-physiological parameters differed significantly between the EMTW and control irrigation. These findings motivate further exploration of molecular mechanisms potentially involved in these effects. DFT simulations suggest that EM treatment may influence water by restructuring its hydrogen-bond network.

Two factors may contribute to enhanced leaf gas exchange under EMTW. First, a modest increase in chlorophyll was observed. Second, several studies report increases in essential nutrients (N, P, K, Ca, Mg) in plants irrigated with electromagnetically treated water [19,20], which could support photosynthetic capacity. However, since the chlorophyll concentration increases by only about ~9%, other factors likely contributed to the improvement in photosynthesis—e.g., enhanced ion transport, changes in quantum yield, and altered cellular redox status. Similar responses have been reported in wheat irrigated with EMTW [19,20,35]. Across crops, magnetically activated water has also been associated with higher net photosynthetic rate and water-use efficiency, alongside gains in quantum yield, light-saturation point, and photosynthetic capacity [36,37,38,39].

Electromagnetically treated water has been reported to enhance nutrient transport, photosynthetic pigments, and flavonoids in maize, with concurrent gains in photosynthetic rate, but the flavonoid content (Table 4) is also consistent with these reports. Similar results showed significantly higher levels of quercetin, rosmarinic acid, and ferulic acid, as measured via HPLC, in the magnetically treated group compared to the control, alongside enhanced antioxidant activity [32,40]. Irrigation of grapevine plants with magnetically treated solutions has resulted in a 75% increase in the total flavonoid content (from 3.21 mg QE/g FW to 5.62 mg QE/g FW), as determined by the AlCl_3_ method (TFC). The result with phenolic content has a 63% increase (from 5.47 mg GAE/g to 8.94 mg GAE/g FW), measured using the Folin–Ciocalteu method (TPC), compared to the control plants. Magnetically activated water influences not only growing plants, but can also be used as a pretreatment on seeds indirectly. Seeds soaked in water and then exposed to electromagnetic waves showed a 52.7% increase in total flavonoid content and an 85% increase over the non-treated control group [41].

Magnetically and electromagnetically treated waters have been used to reduce soil electrical conductivity compared with control irrigation [36]. According to [42], the electromagnetic treatment of saline irrigation water significantly enhanced salt leaching and soil moisture retention, creating a more favorable environment for growth under salinity.

The study extends prior work [43,44] by demonstrating that EMTW effects extend beyond soil improvement, showing that electromagnetically treated water exerts effects beyond soil improvement to encompass molecular- and plant-level responses. Using NMR and DFT, we provide evidence for structural changes and enhanced molecular polarization of water, which are associated with physiological enhancements (*r* = 0.95–0.99). By integrating soil-level context with molecular-level insights, EMTW emerges as a sustainable, multifaceted tool for improving crop performance.

Our DFT results indicate that the EM treatment promotes reorganization of hydrogen bonding and water cluster populations, with an increased number of hydrogen bonds, higher chemical shifts (δ), and higher dipole moment (μ). These observations are consistent with analyses such as those of Lin et al. [45]. In this analysis, EM fields were linked to hydration shell reorganization via altered proton transfer dynamics and molecular orientation: GIAO/DFT calculations (MPW1PW91/6-311+G(2d,p)//MP2/CBS-e) provide molecular/energetic support. The connection between theoretical DFT calculations and properties of bulk water was shown in [28]. Such configurations may influence bulk properties (e.g., surface tension or diffusivity) and, in turn, physiological indicators such as photosynthesis and gas exchange in EMTW-irrigated spinach [7]. This restructuring likely facilitates more efficient water and ion transport and cellular hydration, consistent with the increased net photosynthesis, chlorophyll content, and other indicators observed in spinach under EMTW.

## 4. Materials and Methods

### 4.1. Standardized Irrigation Water

The control and electromagnetically treated water samples had the same physicochemical composition, as verified before treatment. The only experimental variable introduced was the application of EM fields, ensuring that all the observed effects are attributable solely to the field-induced structural modifications of water. The water for irrigation corresponds to Bulgarian Ordinance No. 18 of 27 May 2009, which regulates the quality of water for irrigation of agricultural crops [24,25].

The accredited laboratory Eurotest Control, Sofia, Bulgaria, analyzed the water for irrigation according to the EU standards and reported the following parameters: pH = 7.12 ± 0.1; electrical conductivity σ = 511 ± 5.1 µS·cm^−1^. The EMTW possesses the following parameters: pH = 7.21 ± 0.1; electrical conductivity σ = 536 ± 5.4 µS·cm^−1^.

The measurements were performed at 298.15 K.

### 4.2. Method for Electromagnetically Treated Water (EMTW)

An electromagnetic treatment system was applied using a solenoid system, operating in the ranges of 100–1000 Hz and 10–100 kHz. The treatment system was powered by a 230 W power supply. Alternating current (AC) was applied to two air-core solenoids. The current density J = 3.8 A·mm^−2^ was based on the conductor cross-section area.

The initial experiments were performed with a solenoid [23].

**Figure 6:**
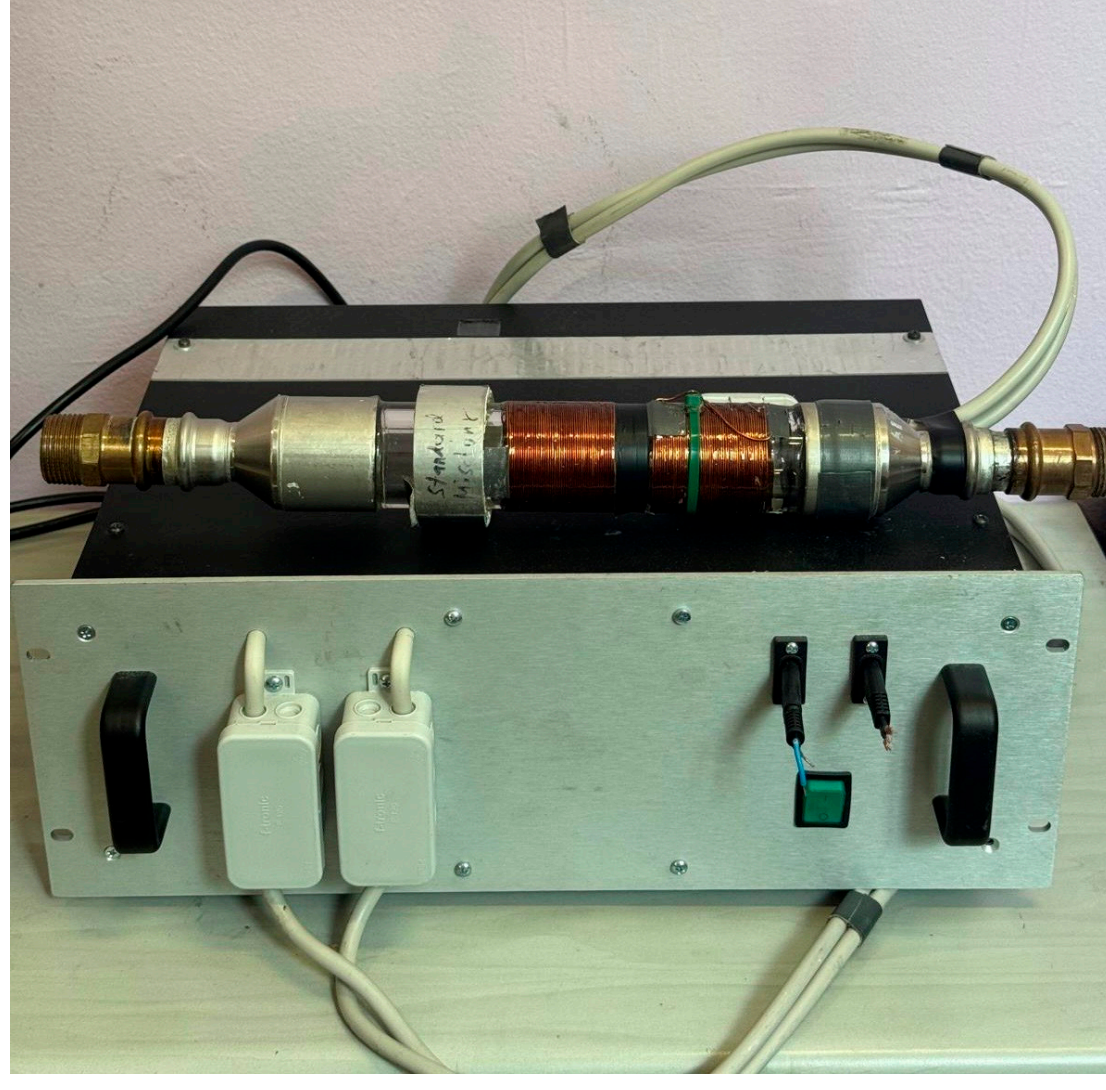
Device with a solenoid for electromagnetically treated water.

Figure 6 demonstrates the device with a solenoid for electromagnetically treated water.

**Figure 7:**
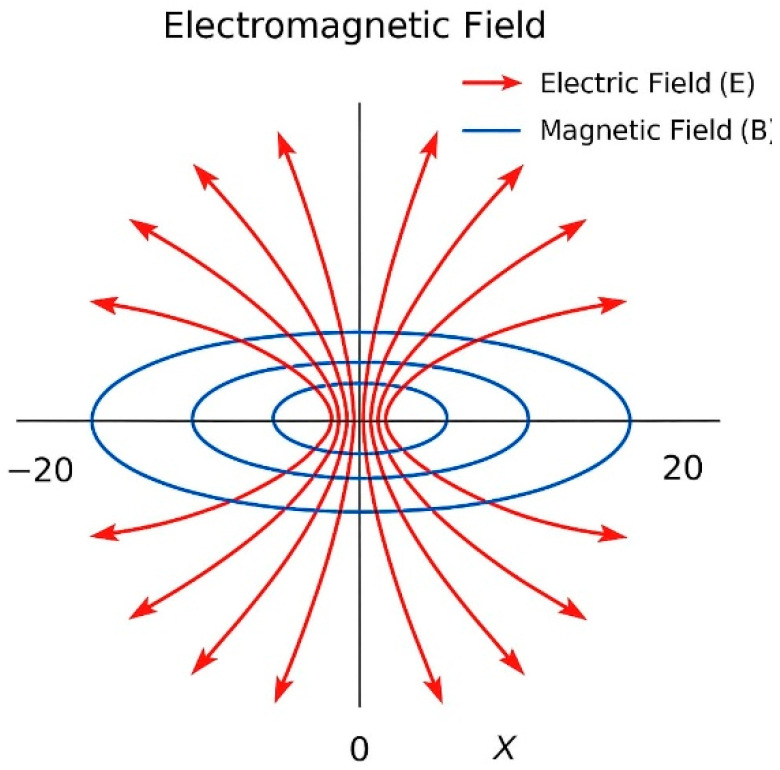
Electromagnetic field structure of the solenoid for EMTW.

Figure 7 shows the electric field (E) radiating outward, while the blue loop indicates the magnetic field (B) circulating around the solenoid axis.

At frequencies (10–100 kHz), significant electrokinetic effects are observed in water, and the operating frequency of 20–40 kHz falls within this range [46,47]. The frequency range of the water treatment device (100–1000 Hz) aligns with findings from previous studies, which show that low-frequency EM waves can influence the microbiological properties of water [23,48].

The plants were irrigated with EMTW while the treatment device operated continuously during the irrigation process.

### 4.3. Nuclear Magnetic Resonance (NMR)

Nuclear magnetic resonance (NMR) spectra were recorded on a Bruker Avance II+ 600 MHz spectrometer (Billerica, MA, USA) with a 5 mm direct detection dual broadband probe. All the experiments were conducted at a temperature of 298 K. Proton (1H) NMR spectra were acquired with 128 K domain points, a spectral width of 9600 Hz, 16 scans, and a relaxation delay of 60 s. Chemical shifts were referenced to a residual signal of dmso-d6 (2.50 ppm), which was placed in a coaxial capillary inside the NMR tube and also served as the lock solvent. The signal half-widths (Δν_1/2_) were determined using the dominant (commanding) peak in each spectrum.

In our research, the water samples were measured with NMR [26,27,28].

### 4.4. Theoretical Calculations for Water Clusters and Hydrogen Bonds

The NMR parameters of the water samples were computed using the Gaussian 16 software package [49]. Structural optimization of the water clusters was carried out using various quantum chemical approaches, including MP2/CBS-e [50], M06-2X/aug-cc-pVDZ [51], by B3LYP/6-31++G** [52], and MP2/aug-cc-pVTZ [53]. In addition, the hybrid density functional MPW1PW91 was employed in conjunction with the 6-311+G(2d,p) basis set, which has been shown to provide reliable predictions for both NMR shielding tensors and geometrical parameters in hydrogen-bonded systems of moderate size [54,55], while maintaining computational efficiency. NMR shielding tensors were calculated using the Gauge-Including Atomic Orbital (GIAO), as implemented in *Gaussian 16*, following the formalism originally introduced by Ditchfield and Hameka [56]. Solvent effects were incorporated implicitly using the Solvation Model based on Density (SMD) with default water parameters, allowing more realistic modeling of the aqueous environment [57].

### 4.5. Plant Material

Spinach plants (*Spinacia oleracea* L.) from both the experimental and control groups were irrigated over six weeks. The first group of plants was grown under field conditions, and the other plants were grown in a laboratory under controlled conditions (air humidity 65%, temperature 22/16 °C, light intensity 350 µmol m^−2^ s^−1^, and photoperiod 12/12).

Within each cultivation condition (field and laboratory), two irrigation treatments were applied per week. The first treatment was with control water (treatment A), and the second treatment was with electromagnetically treated water (treatment B).

Leaf gas exchange (net photosynthetic rate (A_net_), transpiration (E), intercellular concentration of CO_2_ (C_i_), and stomatal conductance (g_s_)) was measured with an open photosynthetic system LCA-4 (Analytical Development Company Ltd., Hoddesdon, UK), equipped with a narrow chamber.

The total chlorophyll content was measured with the CCM-300 Chlorophyll Content Meter (ADC BioScientific Ltd., Hoddesdon, Hertfordshire, UK). The CCM-300 is a hand-held instrument that measures the ratio between the chlorophyll fluorescence at 735 nm and 700–710 nm non-destructively. This ratio is linearly proportional to chlorophyll concentrations as high as 675 mg m^−2^, with a determination coefficient higher than r^2^ = 0.95.

The analyses of leaf gas exchange and chlorophyll content were performed 7 days after the first treatment with magnetically activated water.

### 4.6. Three-Dimensional Analysis of Net Photosynthesis (A_net_), Stomatal Conductance (g_s_), Transpiration (E), and Intercellular CO_2_ Concentration (C_i_)

To analyze the relationship between A, g_s_, E, and C_i_, a three-dimensional (3D) visualization was constructed based on measured data. Physiological measurements of A_net_, g_s_, E, and ci were used as variables for multivariate correlation analysis and model-based surface rendering.

Linear regression models were fitted using the scikit-learn library in Python 3.11. These models treat the dependent variable and g_s_, E, and C_i_ as the independent variables. Surface plots were generated on structured grids with a 20 × 20 resolution and overlaid on top of the measured points to present the predicted physiological behavior. All the 3D graphs were rendered using matplotlib’s Axes 3D toolkit. Color gradients correspond to predicted A values, and the model surfaces were plotted semi-transparently to allow for visual comparison with empirical data.

### 4.7. Analysis of Total Phenols and Flavonoid Content

#### 4.7.1. Plant Extraction Procedure

One gram of a precisely cut sample was soaked in a tube with 10 mL of 70% acidic methanol. The tubes were placed in an ultrasonic bath for 30 min and then kept at room temperature in the dark for 24 h of extraction. After the extraction, the samples were centrifuged at 6708 g for 10 min, and the supernatant was used for analysis of total phenols and flavonoids.

#### 4.7.2. Total Phenol Content Analysis

The total phenol content was measured spectro-photometrically using a UV/Vis spectrophotometer (Spectroquant Pharo300, Merck KGaA, Darmstadt, Germany) at a wavelength of 760 nm. The Folin–Ciocalteu reagent was used to develop the colored complex, following the method of Singleton and Rossi [58], with slight modifications as described by Koleva-Valkova et al. (2017) [59]. Gallic acid served as the standard, and the results were calculated based on a standard curve, expressed as milligrams of Gallic acid equivalents per gram of sample fresh weight.

#### 4.7.3. Total Flavonoid Content Analysis

The amount of total flavonoids was determined by a spectrophotometer “Spectroquant Pharo300”, Merck KGaA, Darmstadt, Germany, at a wavelength of 510 nm according to the method of Zhishen et al. (1999) [60,61], with aluminum trichloride. Quercetin was used to draw a standard curve, and the amount of flavonoids is presented as mg quercetin equivalents per gram of sample fresh weight.

The analyses of phenols and flavonoids content were performed 7 days after the first treatment with magnetically activated water.

## 5. Conclusions

This research investigates the process of water treatment with electromagnetic fields (EMTW) and the responses of *Spinacia oleracea* L. irrigated with EMTW:

1. EMTW exhibits a measurable increase in chemical shift (δ, ppm) and broader resonance peaks (Δν_1/2,_ Hz) in ^1^H-NMR spectroscopy. Complementary DFT simulations show the structuring of the hydrogen network and water cluster with higher parameters according to the control sample.

2. Cluster modeling suggests EMTW promotes aggregation consistent with an enhanced hydrogen-bond network, linked to higher stomatal conductance, CO_2_ assimilation, and chlorophyll content, supporting more efficient gas exchange and photosynthesis.

3. Spinach plants irrigated with EMTW under field and laboratory conditions demonstrated statistically significant improvements in physiological indicators: photosynthesis (A_net_): 80%; transpiration (E): 49–67%; stomatal conductance (g_s_): 78–129%; intercellular CO_2_ concentration (ci): 42–80%; and chlorophyll content: 9.3–9.5%.

4. Pearson’s correlated results were analyzed with strong dependencies between photosynthesis (A_net_) and stomatal conductance (g_s_): r = 0.983; photosynthesis (A_net_) and transpiration (E): r = 0.992.

This reflected a coordinated improvement in both carbon assimilation and water transport, indicative of an optimally tuned physiological system influenced by the properties of EMTW.

5. Under both the field and laboratory conditions, irrigation with EMTW produced stable improvements in agro-physiological traits. The measurable changes in water hydrogen bonding and clustering likely contribute to these effects and future long-term agronomic potential.

## Figures and Tables

**Table 1:** NMR results: δ, ppm, and Δν_1/2_ for electromagnetically treated water and control sample, filtered tap water.

Sample	δ, ppm	Δν_1/2_, Hz	Comment
1	4.192	7.09	Control sample irrigation water
2	4.253	7.68	Electromagnetically treated irrigation water

**Table 2:** Effects of irrigation with EMTW water on physiological indicators in *Spinacia oleracea* L. grown under two types of conditions: laboratory (LG) and field (FG). The control plants (A) are irrigated with untreated water. The experimental group plants (B) are irrigated with electromagnetically treated water.

Plant Growth Regime	Treatments	A_net_	E	g_s_	C_i_	Chlorophyll
LG	A	6.28	0.57	0.07	258	147
	B	11.41	0.95	0.16	284	161
		*p* < 0.001	*p* < 0.001	*p* < 0.01	*p* < 0.01	*p* < 0.01
		r = 0.744	r = 0.640	r = 0.510	r = 0.810	r = 0.712
FG	A	6.22	0.49	0.09	197	139
	B	11.20	0.94	0.16	280	152
		*p* < 0.001	*p* < 0.001	*p* < 0.001	*p* < 0.001	*p* < 0.01
		r = 0.612	r = 0.389	r = 0.481	r = 0.612	r = 0.602

A_net_—net photosynthesis (µmol CO_2_·m^2^·s^−1^); E—transpiration (mmol H_2_O·m^−2^·s^−1^); g_s_—stomatal conductance (mol H_2_O·m^2^·s^−1^); C_i_—intercellular concentration of CO_2_ (µmol·mol^−1^); chlorophyll—concentration (mg·m^−2^); data are average from measurements of 15 *Spinacia oleracea* L. plants from the experimental group and 15 from the control group.

**Table 3:** Correlations between physiological parameters (Pearson).

	r
A (photosynthesis) ↔ E (transpiration)	0.992
A (photosynthesis) ↔ g_s_ (stomatal conductance)	0.983
E (transpiration) ↔ g_s_ (stomatal conductance)	0.952

**Table 4:** Effects of EMTW irrigation on total phenolic and flavonoid contents in field-grown spinach (FG). Control plants (A) were irrigated with untreated water; experimental plants (B) received EMTW.

Plant Growth Regime	Treatments	mgGAE·g^−1^ FW	mgQ·g^−1^ FW
FG	A	2.68	1.03
	B	2.88	1.11
		*p* < 0.001	*p* < 0.001

Total phenolic content expressed as gallic acid equivalents (mg GAE·g^−1^ FW); total flavonoid content expressed as quercetin equivalents (mg QE·g^−1^ FW). Data are means of 15 *Spinacia oleracea* L. plants from each group.

## Data Availability

The original contributions presented in this study are included in the article. Further inquiries can be directed to the corresponding author. The data are not publicly available due to privacy and ethical reasons.

## Abbreviations

The following abbreviations are used in this manuscript:

EMTW	electromagnetically treated water
EM	electromagnetic
MTW	magnetically treated water
NMR	nuclear magnetic resonance
DFT	density functional theory
FG	field-grown
LG	laboratory-grown
A_net_	net photosynthesis
E	transpiration
g_s_	stomatal conductance
C_i_	intercellular concentration of CO_2_
